# New Books

**Published:** 2006-10

A Theory of General Ethics: Human Relationships, Nature, and the Built Environment

Warwick Fox

Cambridge, MA:MIT Press, 2006. 400 pp. ISBN: 0-262-06255-0, $68

Analyzing International Environmental Regimes

Helmut Breitmeier, Oran R. Young, Michael Zürn

Cambridge, MA:MIT Press, 2006. 336 pp. ISBN: 0-262-02602-3, $67

Bilateral Ecopolitics

Philippe Le Prestre, Peter Stoett

Williston, VT:Ashgate Publishing, 2006. 306 pp. ISBN: 0-7546-4177-5, $99.95

Environmental Health in Central and Eastern Europe

K.C. Donnelly, Leslie H. Cizmas, eds.

New York:Springer, 2006. 249 pp. ISBN: 1-4020-4844-0, $119.00

Environmental Principles and Ethics

Ming H. Wong, Frank W.K. Lee, Martin K.F. Fung

Hackensack, NJ:World Scientific Publishing Co., 2006. 240 pp. ISBN: 981-256-838-7, $48

Governing Environmental Flows

Gert Spaargaren, Arthur P.J. Mol, Frederick H. Buttel, eds.

Cambridge, MA:MIT Press, 2006. 392 pp. ISBN: 0-262-69335-6, $27

Governing Global Desertification: Linking Environmental Degradation, Poverty and Participation

Pierre Marc Johnson, Karel Mayrand, and Marc Paquin

Williston, VT:Ashgate Publishing, 2006. 312 pp. ISBN: 0-7546-4359-X, $99.95

Noxious New York: The Racial Politics of Urban Health and Environmental Justice

Julie Sze

Cambridge, MA:MIT Press, 2006. 296 pp. ISBN: 0-262-19554-2, $60

Precautionary Politics: Principle and Practice in Confronting Environmental Risk

Kerry H. Whiteside

Cambridge, MA:MIT Press, 2006. 208 pp. ISBN: 0-262-23255-3, $50

Sustainability, Civil Society and International Governance

John J. Kirton, Peter I. Hajnal

Williston, VT:Ashgate Publishing, 2006. 422 pp. ISBN: 0-7546-3884-7, $114.95

The Environment in Asia Pacific Harbours

Eric Wolanski

New York:Springer, 2006. 497 pp. ISBN: 1-4020-3654-X, $209

